# FBXW7 Reduces the Cancer Stem Cell-Like Properties of Hepatocellular Carcinoma by Regulating the Ubiquitination and Degradation of ACTL6A

**DOI:** 10.1155/2022/3242482

**Published:** 2022-09-14

**Authors:** Xing Wang, Ying Li, Yongning Li, Peng Liu, Songbai Liu, Yaozhen Pan

**Affiliations:** College of Clinical Medicine, Guizhou Medical University, Guiyang, 550000 Guizhou, China

## Abstract

Cancer stem cells (CSCs) comprise a subset of tumor cells that can initiate tumorigenesis and promote tumor advance. A previous study showed that the expression of FBXW7 in hepatocellular carcinoma (HCC) clinical samples was lower than that in the adjacent nontumor tissues and was negatively correlated with the invasion and migration of HCC cells. However, the biological characteristics and the underlying molecular mechanisms of FBXW7 in HCC stemness are yet to be elucidated. In present study, we found that FBXW7 participates in the self-renewal, tumorigenicity, sorafenib therapy, and stem cell-like properties of HCC cells *in vivo* and *in vitro*. The upregulation of FBXW7 inhibited the stemness and reduced the tumorigenicity and drug resistance of HCC cells. Mechanistically, proteins binding to FBXW7 were identified by coimmunoprecipitation and protein colocalization assays. We confirmed ACTL6A as a novel downstream target for FBXW7. The *in vivo* ubiquitination assay showed that FBXW7 repressed HCC malignancy by regulating the oncogenic activity of ACTL6A in a ubiquitin-dependent manner. Furthermore, we found that ACTL6A overexpression inversed the self-renewal abilities and tumorigenic abilities depressed by overexpressing FBXW7. The current findings suggested that FBXW7 reduces the stemness of HCC cells by targeting and degrading ACTL6A and provides a novel target for the diagnosis and treatment of HCC.

## 1. Introduction

Hepatocellular carcinoma (HCC) is one of the most common malignant tumors. It causes >905000 new cases and 830000 deaths annually, making it the sixth most common cancer and the third leading cause of cancer-related mortality worldwide [1]. With the development of liver transplantation, radiotherapy, chemotherapy, and targeting technology, the clinical prognosis of liver cancer has improved [2–4], whereas the poor prognosis of HCC is caused by the advanced tumor and distant metastasis or recurrence. The lack of therapeutic options and hepatic complications underlie the limited treatment of advanced HCC [5, 6]. Hence, effective diagnosis and therapeutic strategies to prevent HCC metastases and relapses are urgent requirements.

Cancer stem cells (CSCs) are a small proportion of tumor cells that can maintain differentiation and self-renewal [7]. They have specific phenotypes, including tumorigenic potential and resistance to radiotherapy and chemotherapy, that are the main causes of distant metastasis and relapse [8, 9]. CSCs have been isolated and identified based on the stem cell-like properties and cell surface markers in many tumors, including HCC [10–12]. Previous studies indicated that targeting CSCs may be an effective approach in anticancer therapy [13, 14]. Therefore, an in-depth study of the molecular mechanisms underlying the phenotypic and functional heterogeneity of CSCs would be useful in developing novel therapeutic strategies against tumor metastasis and relapse.

Accumulating evidence suggested that many abnormally regulated genes or pathways are related to HCC progression [15, 16]. The ubiquitin-proteasome pathway involves a cascade of three enzymes, catalyzed by the ubiquitin-activating enzyme E1, and successively recognized and bound to the target protein by the ubiquitin-conjugating enzyme E2 and ubiquitin ligase E3 [17]. These enzymes determine an array of protein functions. Moreover, the ubiquitin-proteasome pathway plays a vital regulatory role in posttranslational modification, stem cell differentiation, and tumor initiation and metastasis [18–20]. F-box and WD repeat domain-containing 7 (FBXW7) is a member of the E3 family of ubiquitin ligases and has been downregulated in various human cancers, including HCC, colon cancer, and breast cancer [21–23]. It is a tumor suppressor and positively correlated with the clinical prognosis of patients [24]. Several studies have shown that FBXW7 targets a series of key oncoproteins, such as c-Myc, cyclin E, mTOR, and Notch1 [25, 26]. In a previous study, we showed that FBXW7 inhibits HCC migration and invasion by targeting the Notch1 signaling pathway in HCC [27]. To date, FBXW7 has been shown to regulate cell differentiation, progenitor cell apoptosis, and cell cycle quiescence, but its mechanism in CSCs, including liver cancer stem cells (LCSCs), is not yet elucidated [28, 29]. Thus, revealing the molecular mechanism of abnormal expression of FBXW7 is crucial to exploring the pathogenesis of HCC.

Actin-like 6A (ACTL6A) is a member of adenosine triphosphate-dependent SWI/SNF-like BRG1/BRM-associated factor (BAF) chromatin remodeling complexes. The BAF complexes are implicated in epigenetic regulators of the chromatin structure [30, 31]. Recent studies revealed that BAF complexes are essential for pluripotency in the regulation of embryonic and adult stem cell function [32, 33]. However, BAF complexes are composed of 11 subunits encoded by multiple actin-like genes associated with various epigenetic regulators. Whether these actin-like genes participate in regulating the biological function of CSCs is not yet clarified. Some genes or pathways affected by ACTL6A, such as *p53*, sex-determining region Y-box 2 (*SOX2*), epithelial-mesenchymal transition (EMT), and Notch and YAP signaling pathway, maintain the CSC-like properties [34, 35]. Based on the integrated analyses, we hypothesized that ACTL6A has a specific effect on the metastasis and relapse of HCC and phenotypic regulation of CSCs.

Herein, we investigated whether FBXW7 is involved in the malignant phenotype of HCC cells and found that it could inhibit the stemness and malignancy in HCC in both *in vivo* and *in vitro* studies. We also demonstrated that ACTL6A was a potential target of FBXW7, and the overexpression of FBXW7 significantly inhibited stem cell-like properties by disrupting the stability of the ACTL6A protein via the ubiquitin-proteasome pathway. Together, these findings showed that FBXW7 acts as an ACTL6A ubiquitinase, making it a potential target for therapy of advanced HCC. We also confirmed that targeting ACTL6A may be a molecular therapy to prevent metastasis or recurrence by reducing the CSCs in the HCC microenvironment.

## 2. Materials and Methods

### 2.1. Reagents and Cell Culture

All the antibodies were purchased from Proteintech (Wuhan, China) except FBXW7 (ab109617, Abcam, Cambridge, MA, USA) and CD133-PE (130-113-670, Miltenyi Biotec, Bergisch Gladbach, Germany). Cycloheximide (CHX) and MG132 were purchased from MedChemExpress (Shanghai, China). The HCC cell lines MHCC-97H and Huh7 were purchased from the Cell Bank of the Chinese Academy of Sciences (Shanghai, China). The two cell lines were cultivated in Dulbecco's modified Eagle's medium (DMEM, Gibco, Grand Island, USA) supplemented with 10% fetal bovine serum (FBS, Gibco) and 1% penicillin/streptomycin (Gibco) at 37°C in 5% CO_2_.

### 2.2. Construction of Lentiviral Vectors and Stable Cell Lines

Flag-tagged FBXW7 and ACTL6A constructs were generated by inserting the corresponding coding sequence into the lentiviral vector GV341 (GeneChem, Shanghai, China). MHCC-97H and Huh7 cells with stably overexpressed FBXW7 or ACTL6A constructs were established. Furthermore, the target sequence corresponding to FBXW7 or ACTL6A (FBXW7-shRNA#1: 5′-GGAACCCAAAGACCTGCTA-3′, FBXW7-shRNA#2: 5′-GTTAGTGGTTCTGATGAC A-3′, ACTL6A-shRNA#1: 5′-TGCTTCCCTCCTTCTTCA-3′, ACTL6A-shRNA#2: 5′-CATCGTGGACTGGAATTGCAG-3′) was cloned into the lentiviral vector GV112 to establish a stable FBXW7 or ACTL6A knocked down cell line. Lentivirus and GP solution was used to infect HCC cells. At 48 h postinfection, the cells were cultured in a DMEM medium containing 5 *μ*g/mL puromycin for 7 days. The efficiency of overexpression and knockdown was analyzed by quantitative real-time polymerase chain reaction (RT-qPCR) and Western blot.

### 2.3. RNA Extraction and RT-qPCR

Total RNA was extracted using TRIzol reagent (Invitrogen, Waltham, MA, USA), and the concentration and quality of isolated RNA were detected by the spectrophotometer (NanoDrop™ One/OneC, Thermo Scientific). Complementary DNA (cDNA) was generated from mRNA using oligo-dT primers and PrimeScript RT reagent kit (Takara, Beijing, China). The cDNA was used as a template in RT-qPCR using TB Green Premix ExTaq (Takara) on the CFX96™ Real-Time system (Bio-Rad, CA, USA). *GAPDH* was used as an internal control, and the target gene expression was calculated by the 2^-*ΔΔ*Ct^ method. The primer sequences are listed in Additional File 1.

### 2.4. Western Blot and Coimmunoprecipitation (IP)

The cells were lysed with RIPA buffer consisting of phosphatase and protease inhibitors on ice for 10 min. Next, the cells were sonicated by ultrasonication for 2 min. The supernatant was collected by centrifugation at 12000 rpm 4°C for 15 min. Proteins were separated by 10% SDS-PAGE and transferred to the polyvinylidene fluoride (PVDF) membrane. Then, the membrane was probed with primary antibodies, such as FBXW7 (1 : 1000), ACTL6A (1 : 1000), GAPDH (1 : 10000), and anti-HA (1 : 1000). The Image Lab software was used for quantitative analysis of protein expression. The IP extracts were prepared using NP-40 lysis buffer and incubated with the corresponding antibody at 4°C overnight. Subsequently, the extract was incubated with protein A/G beads (Beyotime, Shanghai, China) at 4°C for 4 h. Finally, the bead coupling complex was analyzed by Western blot.

### 2.5. Flow Cytometry

For detection of the CD133 proportion, cells were washed with PBS by centrifugation and incubated with CD133-PE antibody (1 : 50) at 4°C for 10 min in the dark. Subsequently, the cells were washed with 1 mL buffer by centrifugation at 300×*g* for 10 min. Finally, the cells were resuspended in a 500 *μ*L buffer for analysis. To detect the cell cycle distribution, the cells were incubated with 1 mL of DNA staining solution supplemented with 10 *μ*L permeabilization solution (Multi sciences, China) for 30 min in the dark. The cells distribution was detected by flow cytometry (Beckman, CA, USA).

### 2.6. Sphere Formation Assays

The cells were seeded into 6-well ultralow attachment plate (Corning, NY, USA) at a density of 1000 cells/well and cultivated in serum-free DMEM/F12 medium (Invitrogen) supplemented with 2% B27 (Invitrogen), EGF (20 ng/mL; Invitrogen), and bFGF (10 ng/mL; Invitrogen). After culturing for 14 days, the spheres were counted and analyzed with a diameter > 50 *μ*m.

### 2.7. Immunohistochemistry (IHC) and Immunofluorescence (IF)

The levels of FBXW7 and ACTL6A proteins and several pluripotent transcription factors in HCC xenografts were confirmed by IHC. The tissue was fixed in 4% formalin and embedded in paraffin. The sections were incubated with primary antibodies at 4°C overnight and analyzed under the microscope (TE2000-U, Nikon, Japan). For IF, the cells were fixed with 4% formaldehyde and stained with primary antibodies (1 : 200) at 4°C for 8–12 h, followed by incubation with Alexa Fluor 594-conjugated goat anti-rabbit IgG (1 : 200) or Alexa Fluor 488-conjugated goat anti-mouse IgG (1 : 200) for 1 h. Then, cell nuclei were stained with 4′,6-diamidino-2-phenylindole (DAPI) and observed by confocal microscopy.

### 2.8. *In Vivo* Ubiquitination Assay

The cells were grown to 80–90% confluency in a 10 cm dish. The HA-ubiquitin plasmids (GeneChem) were transfected into cells using Lipofectamine 3000, P3000™ (Invitrogen), and Opti-MEM culture medium (Thermo Fisher, Suzhou, China). FBXW7 overexpression cells and control cells (vector), FBXW7 depletion cells (shFBXW7#1), and negative control cells (shNC) were incubated with MG132 (10 *μ*M) for 12 h before harvesting and lysis by NP-40 lysis buffer. Then, the proteins were immunoprecipitated to detect the ubiquitination of ACTL6A using an anti-ACTL6A antibody. The whole-cell extracts were incubated with 10 *μ*L anti-HA magnetic beads (MedChemExpress) at 4°C overnight. Finally, the bead-bound protein complex was eluted in 50 *μ*L of 1X Loading buffer, boiled for 10 min, and detected through the immunoblotting assay.

### 2.9. Tumor Xenografts

For xenograft models, BALB/c-nu mice, aged 4–6 weeks old, were randomly divided into seven groups and subcutaneously inoculated with MHCC-97H LV-vector, LV-FBXW7, LV-ACTL6A, LV-ACTL6A+FBXW7 cells, Huh7 LV-shNC, and LV-shFBXW7#1 cells (5 × 10^6^ cells/200 *μ*L, *n* = 4). The tumor length (*a*) and width (*b*) were assessed once a week, and the tumor volume = *a* × *b*^2^/2 (mm^3^). After observing the growth of xenograft for 6 weeks, the mice were sacrificed, and the weight of the tumors was measured. Then, the tumors were fixed in 4% formaldehyde and analyzed by IHC staining for the indicated antibodies. All experimental procedures were approved by the Institutional Animal Care and Use Committee (IACUC) of Guizhou Medical University.

### 2.10. Statistical Analysis

Statistical analyses were performed using SPSS 25.0 (IBM; SPSS, IL, USA). The graphs were generated using GraphPad Prism 8.0 (GraphPad Software, CA, USA). The data are presented as means ± standard deviation (SD). Student's *t*-test was performed to assess the statistical significance between the two groups. *P* < 0.05 indicated statistical significance.

## 3. Results

### 3.1. FBXW7 Overexpression Inhibits the Stem Cell-Like Properties of HCC Cells

**Figure 1:**
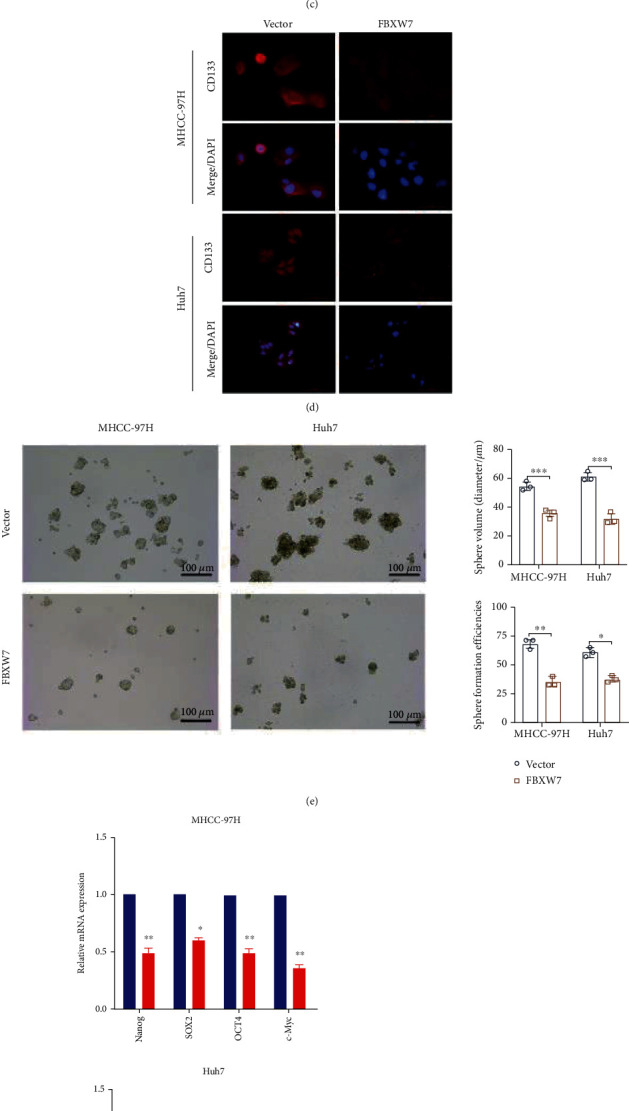
FBXW7 overexpression inhibits the stem cell-like properties of HCC cells. (a) FBXW7 expression was detected in spheres and their adherent HCC cells by RT-qPCR and Western blot. (b) RT-qPCR and Western blot analysis of mRNA and protein level of FBXW7 in the cell lines infected with LV-FBXW7 and LV-Vector. (c, d) CD133 surface marker expression was analyzed in the two cell lines by flow cytometry and IF staining. (e) Representative images and quantitative analysis of spheres. Scale bar: 100 mm. (f, g) Pluripotent transcription factors, including Nanog, SOX2, OCT4, and c-Myc, were analyzed by q-PCR (f) and Western blot (g). (h) Flow cytometry showed the effect of FBXW7 on the cell cycle in MHCC-97H and Huh7 cells. (i) Western blot analysis of cyclin E1 and p21 after FBXW7 alternation in MHCC-97H and Huh7 cells. Graph represents mean ± SD; ^∗^*P* < 0.05, ^∗∗^*P* < 0.01, ^∗∗∗^*P* < 0.001.

Our previous studies indicated that FBXW7 is markedly downregulated in HCC and is related to tumor migration, invasion, and progression [27]. In this study, we enriched the CSCs by inducing tumor sphere formation from HCC cell lines MHCC-97H and Huh7 and measured the mRNA and protein level of FBXW7. FBXW7 was downregulated in tumor spheres compared to their adherent cells (Figure 1(a)).

To detect the impact of FBXW7 on the stemness of HCC cells, the lentiviral FBXW7 was stably infected into the MHCC-97H and Huh7 cells. The empty lentivector was used as a control (Figure 1(b)). Previous studies suggested that one of the sources of CSCs may be mutated from adult stem/progenitor cells in the corresponding organ as they exhibit high expression of stemness-related genes and specific surface markers [36]. The LCSC surface marker CD133 was detected by flow cytometry and IF. These findings showed that FBXW7 overexpression reduces the proportion of CD133+ cells in both cell lines (Figures 1(c) and 1(d)). Next, we assessed the role of FBXW7 in HCC cells self-renewal. As shown in Figure 1(e), the overexpression of FBXW7 suppresses the size and generation of tumor spheres. RT-qPCR and Western blot were conducted to investigate whether FBXW7 affects the expression of stemness-related markers in HCC cells. As expected, FBXW7 overexpression reduced the level of these markers (Figures 1(f) and 1(g)). Finally, the effect of FBXW7 on cell cycle was detected by flow cytometry. As shown in Figure 1(h), the overexpression of FBXW7 decreased the proportion of S phase cells and increased the proportion of G0/G1 phase cells. Western blot results showed that FBXW7 overexpression inhibited the levels of cyclin E1 and increased the levels of p21 protein (Figure 1(i)).

### 3.2. FBXW7 Downregulation Promotes the Stem Cell-Like Properties of HCC Cells

**Figure 2:**
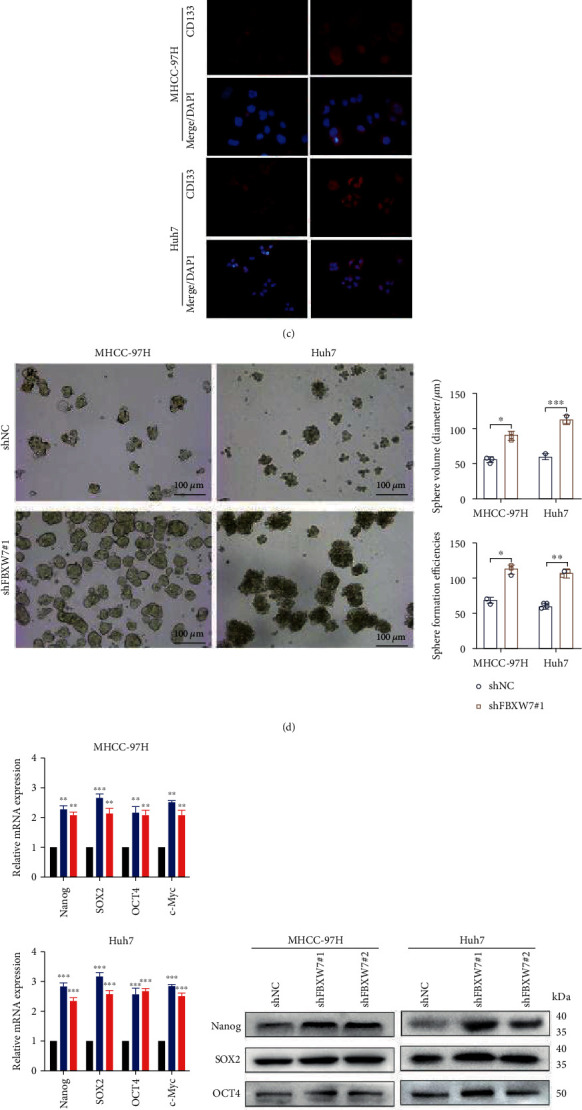
FBXW7 downregulation promotes the stem cell-like properties of HCC cells. (a) RT-qPCR and Western blot analysis of mRNA and protein level of FBXW7 in the cell lines infected with LV-shFBXW7#1, #2, and shNC. (c–h) The experimental method is the same as Figure 1. Graph represents mean ± SD; ^∗^*P* < 0.05, ^∗∗^*P* < 0.01, ^∗∗∗^*P* < 0.001.

To further explore the impact of FBXW7 on the CSC-like properties, two short hairpin RNAs (shFBXW7#1 and shFBXW7#2) exclusively targeting FBXW7 were used to infect the cell lines. RT-qPCR and Western blot showed that FBXW7 expression was downregulated by the shRNA (Figure 2(a)). Next, we chose shFBXW7#1 for the subsequent experiments, and the proportion of CD133 cells increased after FBXW7 knockdown compared to the control group (Figures 2(b) and 2(c)). The sphere formation assay also showed that the downregulation of FBXW7 promoted the size and generation of tumor spheres (Figure 2(d)). The RT-qPCR and Western blot analyzed that FBXW7 knockdown increases the levels of pluripotent transcription factors compared to control cells (Figures 2(e) and 2(f)). FBXW7 downregulation increased the proportion of S phase cells and decreased the proportion of G0/G1 phase cells (Figure 2(g)). Western blot observed that FBXW7 knockdown elevated the expression of cyclin E1 while decreasing the levels of p21 (Figure 2(h)).

### 3.3. FBXW7 Inhibits the Tumorigenesis *In Vivo* and Interferes with Sorafenib Resistance of HCC Cells

**Figure 3:**
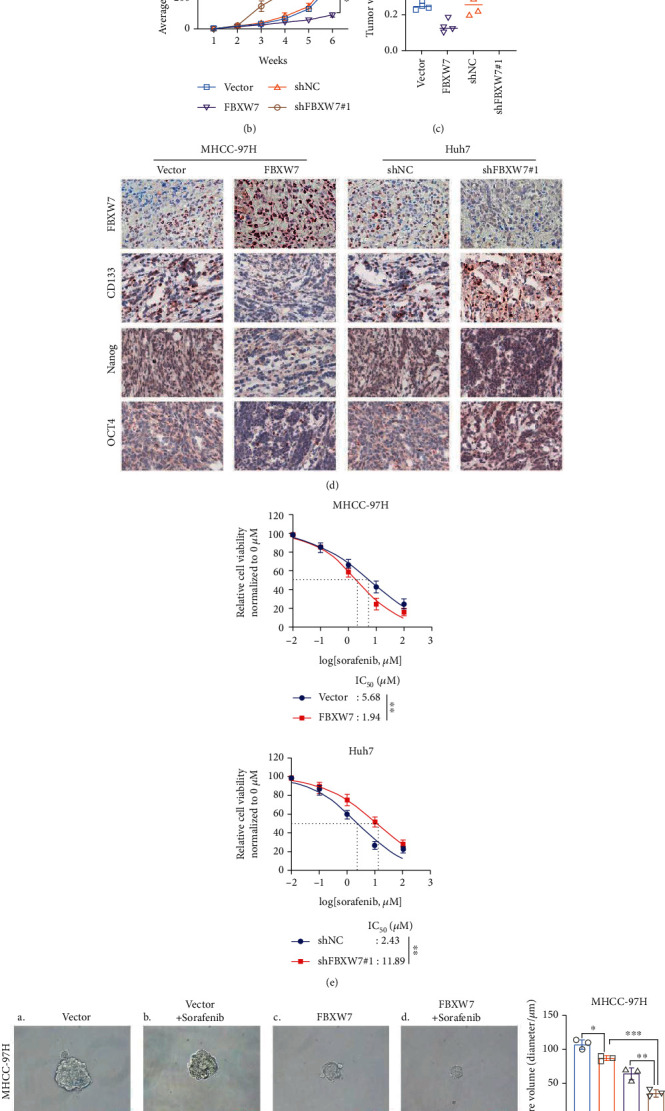
FBXW7 inhibits the tumorigenesis *in vivo* and interferes with sorafenib resistance of HCC cells. (a) Images of xenografts in nude mice 6 weeks after subcutaneous injection of indicated cells. (b, c) Xenograft tumors growth curve and weight. Tumor volume was calculated by (length × width^2^)/2. (d) IHC staining of FBXW7 and pluripotency-associated markers in respective xenograft tumor tissues. (e) FBXW7 overexpression increased the sensitivity to sorafenib, while FBXW7 knockdown reduced the sensitivity to sorafenib. (f) The influence of FBXW7 or sorafenib on the sphere formation in MHCC-97H or Huh7 cells. Graph represents mean ± SD; ^∗^*P* < 0.05, ^∗∗^*P* < 0.01, ^∗∗∗^*P* < 0.001.

To investigate whether FBXW7 interferes with the tumorigenic potential of HCC cells *in vivo*, we selected nude mice to establish a subcutaneous xenograft model. Results showed that FBXW7 overexpression significantly inhibits the growth rate, tumor size, and xenograft weight compared to the vector group, while FBXW7 knockdown showed the opposite trend (Figures 3(a)–3(c)). Consistently, IHC staining showed low expression of cellular stemness-related molecules, such as CD133, Nanog, and OCT4, in FBXW7-overexpressing tumors compared to the vector specimens while high levels of these markers were detected in the xenografts comprising shFBXW7#1-treated cells (Figure 3(d)). This finding confirmed that FBXW7 reduces the CSC marker expression and suppresses HCC cell tumorigenesis *in vivo*.

Since the stem cell-like characteristics are closely associated with tumor recurrence and chemotherapy resistance, we speculated that the expression of FBXW7 may affect the drug sensitivity of HCC cells. Previous studies have shown that sorafenib is the most commonly used targeted anticancer drug for patients with advanced liver cancer or metastasis in addition to the basic chemotherapy regimen. Thus, the cell counting kit-8 (CCK-8) assay was used to analyze whether FBXW7 expression affected the sensitivity of HCC cells to sorafenib. The half-maximal inhibitory concentration (IC_50_) value of sorafenib was significantly decreased in MHCC-97H-FBXW7 cells compared to the vector but significantly increased in Huh7-shFBXW7#1 cells (Figure 3(e)). The sphere formation assay revealed that the self-renewal ability of MHCC-97H cells decreased significantly after FBXW7 overexpression combined with sorafenib intervention. In comparison, the downregulation of FBXW7 weakened the inhibitory effect of sorafenib on the self-renewal of Huh7 cells (Figure 3(f)). Collectively, these findings indicated that FBXW7 inhibits the self-renewal capacity of HCC cells and improves the sensitivity of sorafenib therapy *in vitro*.

### 3.4. E3 Ubiquitin Ligase FBXW7 Interacts with ACTL6A

**Figure 4:**
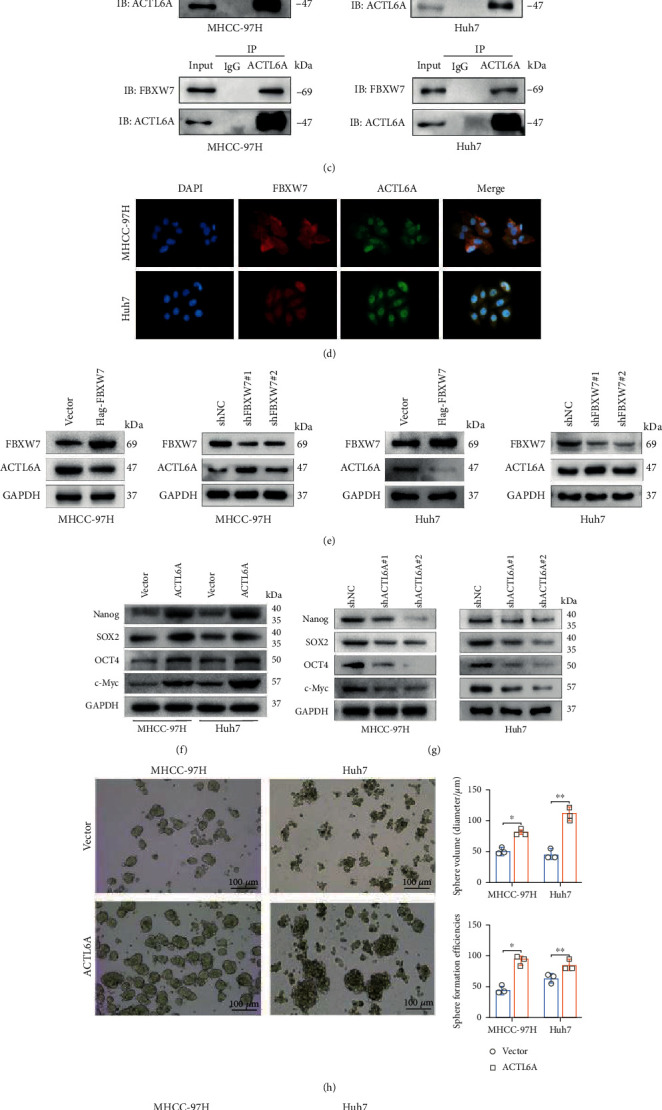
E3 ubiquitin ligase FBXW7 interacts with ACTL6A. (a) Cell extracts from MHCC-97 cells with Flag-FBXW7 expression were immunopurified on an anti-Flag affinity column and eluted with Flag peptides. The eluates were analyzed by mass spectrometry. (b) Predictive analysis of E3 ubiquitin ligases that might target ACTL6A was performed using UbiBrowser database. (c) Co-IP and immunoblotting detected the interaction between FBXW7 and ACTL6A. (d) IF staining of FBXW7 and ACTL6A in the above cells. (e) Western blot was used to detect the effect of FBXW7 on the ACTL6A protein level. (f, g) Comparison of the expression of pluripotency-associated markers in HCC cells of different groups by Western blot. (h, i) Self-renewal abilities were measured by sphere formation assays in MHCC-97H and Huh7 cells transfected with LV-ACTL6A or shACTL6A#2. Graph represents mean ± SD; ^∗^*P* < 0.05, ^∗∗^*P* < 0.01.

To investigate the molecular mechanism underlying the inhibition of FBXW7 on HCC stemness, the subcellular complexes interacting with FBXW7 were detected by IP and mass spectrometry; consequently, several proteins, including ACTL6A, were obtained (Figure 4(a) and Additional File 1). Importantly, FBXW7 is a critical E3 ligase regulating the ubiquitin reaction of ACTL6A through the UbiBrowser database (http://ubibrowser.ncpsb.org.cn) (Figure 4(b)). Next, we examined the physical links between FBXW7 and ACTL6A in the above cell lines by Co-IP assays. The IP with anti-FBXW7 antibody and Western blot analysis of ACTL6A showed that ACTL6A was efficiently coimmunoprecipitated with ectopically expressed FBXW7 (Figure 4(c), top panel). Furthermore, FBXW7 protein was detected when ACTL6A was immunoprecipitated by ACTL6A antibodies (Figure 4(c), down panel). Specifically, the IF staining presented the colocalization of FBXW7 and ACTL6A in both cell lines (Figure 4(d)). These results strongly suggested that FBXW7 interacts with ACTL6A.

### 3.5. FBXW7 Negatively Regulates ACTL6A Stability via the Ubiquitin-Proteasome Pathway

First, we evaluated whether FBXW7 regulated the expression of ACTL6A. As expected, the ACTL6A protein level decreased significantly with the increased FBXW7 expression, while FBXW7 knockdown increased ACTL6A expression (Figure 4(e)). To investigate whether ACTL6A retained the stemness of HCC cells, silencing ACTL6A using two independent shRNAs led to a notable decrease in the stemness-related markers Nanog, SOX2, OCT4, and c-Myc, while the ectopic expression of ACTL6A increased the expression of these markers (Figures 4(f) and 4(g)). Accordingly, the knockdown of ACTL6A inhibited the sphere formation efficiency and sphere sizes, whereas the overexpressing presented an opposite trend (Figures 4(h) and 4(i)).

**Figure 5:**
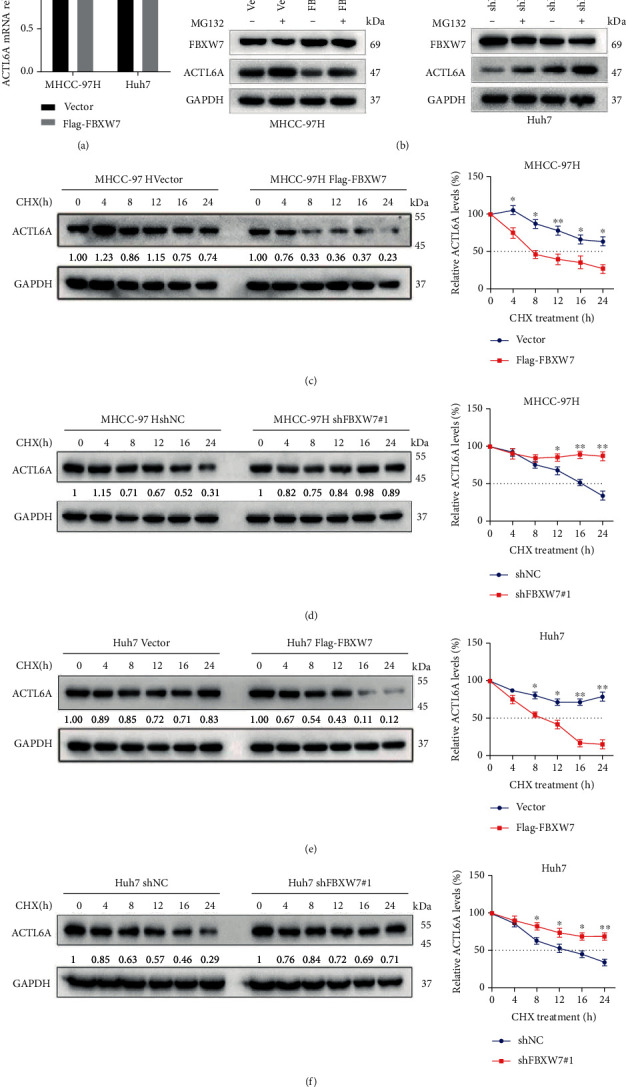
FBXW7 negatively regulates ACTL6A stability via the ubiquitin-proteasome pathway. (a) RT-qPCR analysis of ACTL6A transcript level in MHCC-97H and Huh7 cells. (b) The above cells were incubated with MG132 (10 *μ*M) for 12 h, and total protein was extracted and subjected to Western blot. (c–f) MHCC-97H and Huh7 cells were treated with CHX (50 *μ*g/mL), harvested, and immunoblotted for ACTL6A and GAPDH. Quantification of the ACTL6A protein levels relative to GAPDH expression. Graph represents mean ± SD; ^∗^*P* < 0.05, ^∗∗^*P* < 0.01.

**Figure 6:**
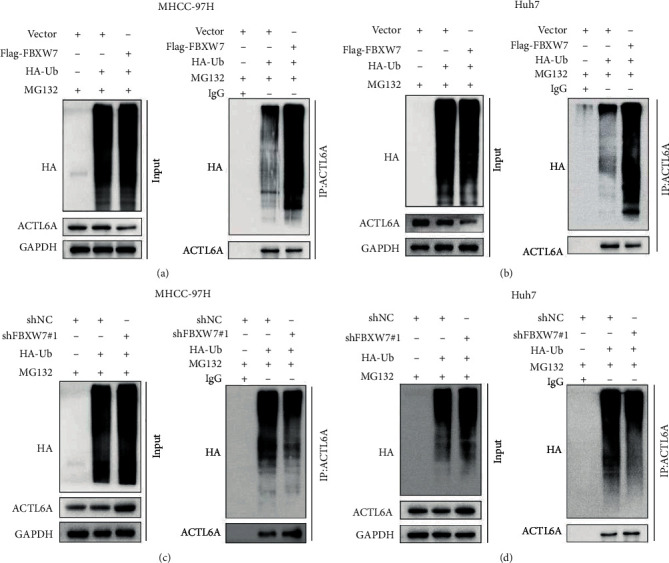
Ubiquitination of ACTL6A in MHCC-97H and Huh7 cells was regulated by FBXW7 overexpression or knockdown in the presence of MG132 (10 *μ*M, 12 h) detected by IP and immunoblotting. (a, c) MHCC-97H cells. (b, d) Huh7 cells.

Furthermore, upregulated FBXW7 had an impact on ACTL6A protein levels but did not affect its mRNA level (Figure 5(a)), indicating that the regulation of ACTL6A by FBXW7 occurs at the posttranscriptional level. Next, we studied whether the ubiquitin pathway regulates the stability of ACTL6A protein. The proteasome inhibitor MG132 showed that MG132 significantly weakens the effect of FBXW7 on ACTL6A (Figure 5(b)). In addition, the cells were treated with CHX for various time points, and the protein extracts were analyzed. We found that the overexpression of FBXW7 in cells shortened the half-life of ACTL6A protein (Figures 5(c) and 5(e)). Conversely, FBXW7 knockdown in cells significantly prolonged the half-life of ACTL6A (Figures 5(d) and 5(f)). The *in vivo* ubiquitination assays investigated whether FBXW7 is essential for the ubiquitination of ACTL6A. The cells were cotransfected with HA-ubiquitin-encoding plasmids with upregulated or silenced FBXW7 and treated with MG132. Subsequently, the cell extract was immunoprecipitated with anti-ACTL6A antibody and detected by immunoblotting with anti-HA antibody. The findings suggested that the overexpression of FBXW7 promotes the ubiquitination of ACTL6A in both cell lines (Figures 6(a) and 6(b)), while FBXW7 downregulation was accompanied by a decrease in the ubiquitination level of ACTL6A (Figures 6(c) and 6(d)). These results further confirmed that the regulation of the target protein might be associated with the ubiquitin-proteasome pathway.

### 3.6. FBXW7 Inhibits HCC Cell Stemness and Cell Malignancy by Downregulating the Levels of ACTL6A

**Figure 7:**
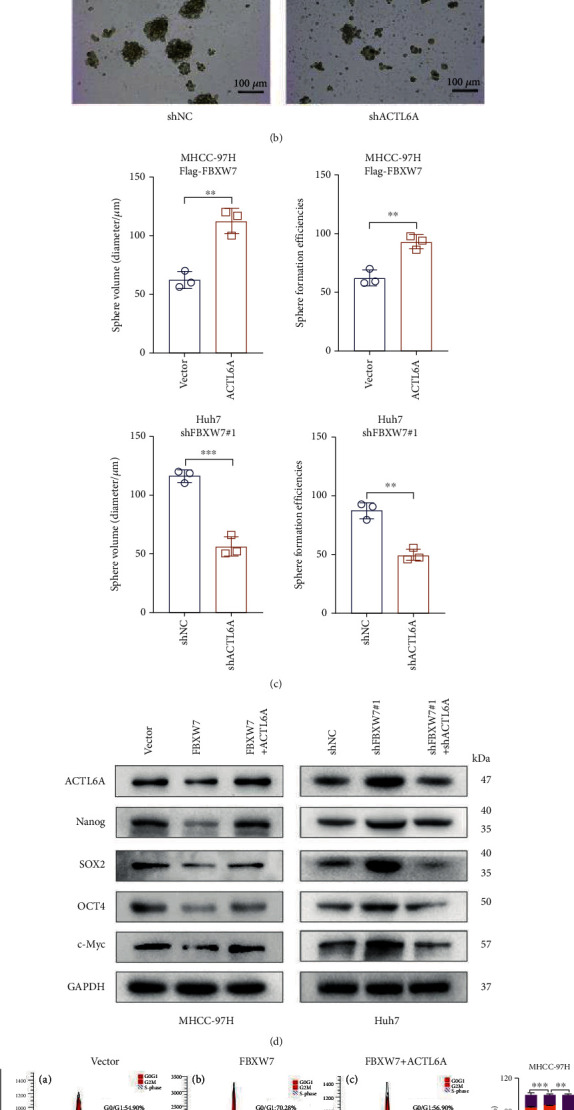
FBXW7 inhibits HCC cell stemness and malignancy by downregulating the levels of ACTL6A. (a) Flow cytometry analysis of the positivity rate of CD133 in different groups. (b, c) Representative images and quantification of the size and generation rate of tumor spheres. Scale bars, 100 *μ*m. (d) Western blot was used to detect the expression of pluripotent transcription factors in HCC cells with FBXW7 or ACTL6A. (e) Representative images and quantification of cell cycle distribution detected by flow cytometry. (f) Expression of cell cycle-related proteins detected by Western blot. Graph represents mean ± SD; ^∗∗^*P* < 0.01, ^∗∗∗^*P* < 0.001.

Based on the above results, we hypothesized that FBXW7 inhibits the stemness of HCC cells through ubiquitination and degradation of ACTL6A. To validate this theory, we reconstituted or disrupted the expression of ACTL6A under FBXW7 overexpression or silencing and detected the changes in stem cell-like properties. Flow cytometry results suggest that ACTL6A overexpression increased the rate of CD133-positive cells in MHCC-97H cells compared to the control group, while the overexpression of FBXW7 reversed this phenomenon (Figure 7(a), top panel). On the other hand, the downregulation of FBXW7 restored the expression of CD133+ marker in ACTL6A-silenced Huh7 cells (Figure 7(a), bottom panel). The FBXW7-mediated inhibition of self-renewal was counteracted by ACTL6A overexpression, as shown in the sphere formation assay, while ACTL6A knockdown abolished the effect of FBXW7-silenced cells (Figures 7(b) and 7(c)). Also, Western blot showed that the pluripotent transcription factors were significantly restored when ACTL6A was upregulated in FBXW7 overexpressing MHCC-97H cells compared to the control cells, whereas ACTL6A knockdown suppressed the protein levels of these factors in FBXW7-silenced Huh7 cells (Figure 7(d)). In addition, we performed the cell cycle recovery experiment and found that ACTL6A restored the inhibition of G1/S phase transition caused by the overexpression of FBXW7. Similarly, FBXW7 knockdown increased the proportion of proliferating Huh7 cells, while ACTL6A downregulation reduced this effect (Figure 7(e)). Consistent with these findings, Western blot showed that FBXW7 overexpression decreased cyclin E1 expression and increased the level of p21, which was reversed after ACTL6A upregulation (Figure 7(f)). Taken together, these findings confirmed that FBXW7 inhibits HCC stemness and cell malignancy by downregulating ACTL6A expression.

### 3.7. FBXW7 Regulates ACTL6A Expression Levels to Inhibit HCC Growth *In Vivo* and Resistance of Sorafenib *In Vitro*

**Figure 8:**
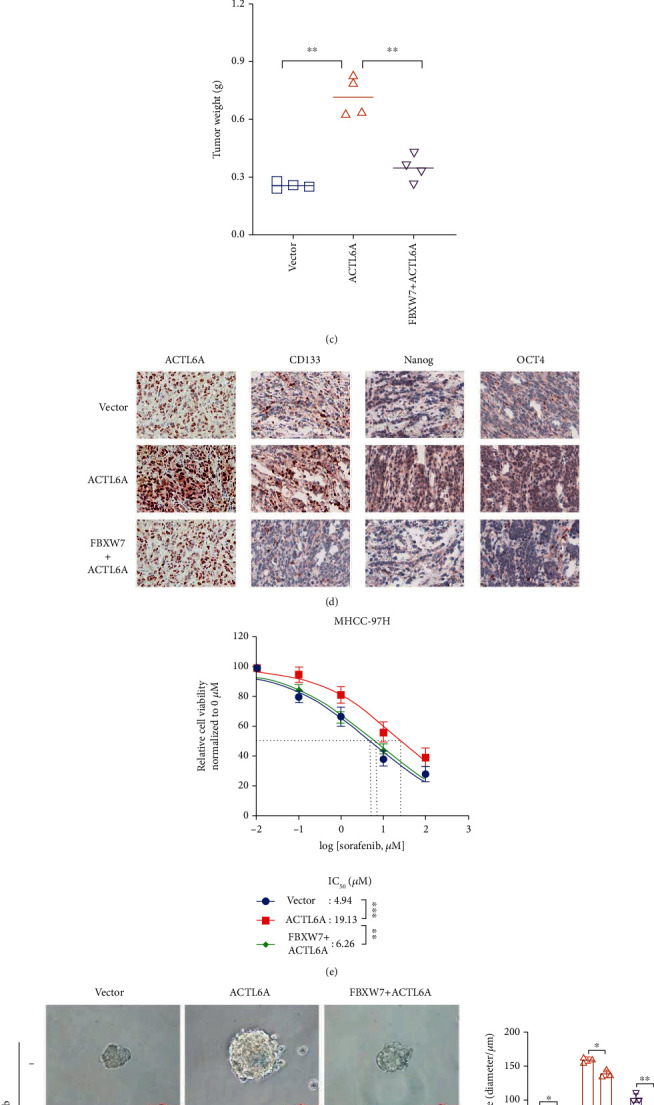
FBXW7 regulates ACTL6A expression levels to inhibit HCC growth *in vivo* and resistance of sorafenib *in vitro*. (a–c) The indicated engineered MHCC-97H or control cells were injected into subcutaneous and representative images of tumors (a), tumor growth curve (b), and tumor weight (c). (d) The expression of ACTL6A, CD133, Nanog, and OCT in xenograft tumor tissues by IHC staining. (e) Overexpression of ACTL6A in MHCC-97H decreased the sensitivity to sorafenib, which was rescued by FBXW7 upregulation. (f) Effect of ACTL6A overexpression combined with sorafenib on tumor spheroid formation in MHCC-97H cells. Graph represents mean ± SD; ^∗^*P* < 0.05, ^∗∗^*P* < 0.01, ^∗∗∗^*P* < 0.001.

To assess the regulation of FBXW7 on ACTL6A *in vivo*, LV-Vector, LV-ACTL6A, and LV-FBXW7+LV-ACTL6A cells were inoculated subcutaneously in nude mice to construct xenograft models. As shown in Figures 8(a)–8(c), ACTL6A overexpression promoted the growth of tumors compared to the control cells. However, FBXW7 reversed the effect caused by ACTL6A. The IHC staining demonstrated that the expression of ACTL6A and pluripotent transcription factors were upregulated in the ACTL6A group compared to the control group. Conversely, the FBXW7+ACTL6A group counteracted all the trends of the ACTL6A group (Figure 8(d)). Furthermore, we explored whether the sensitivity of HCC cells to sorafenib was affected by ACTL6A. The CCK-8 assay indicated that ACTL6A overexpression elevated the IC_50_ value in MHCC-97H cells, whereas upregulated FBXW7 expression showed opposite effects (Figure 8(e)). As shown in Figure 8(f), the sphere formation assay demonstrated that ectopically expressed ACTL6A attenuated the effect of sorafenib on MHCC-97H cells' self-renewal. These results were consistent with the previous findings on the role of ACTL6A as an oncogene in HCC and indicated that FBXW7 inhibits the stemness of tumors by suppressing the functions of ACTL6A.

## 4. Discussion

Despite the continuous progress in the clinical application and systemic treatments of HCC, the disease remains a highly malignant tumor [37, 38]. CSCs represent the subpopulations with stemness properties, such as self-renewal, drug resistance, and tumorigenicity, in cancer cells. Heterogeneity is a feature of CSCs and a major reason affecting tumor malignancy and disease progression [39]. Thus, the existence of CSCs might explain the high recurrence rate postoperatively, the easy metastasis of malignant tumors, and the decrease of sensitivity to radiotherapy and chemotherapy [40, 41]. Previous studies demonstrated that the complete remission of tumors depends on the complete elimination of CSCs, but identifying and targeting CSCs is challenging during the treatment. Thus, the clinically relevant mechanisms underlying the CSC regulation in solid tumors are yet unknown, especially in HCC.

The loss-of-function mutations of FBXW7 are frequently reported in several cancer types [42]. FBXW7 has been reported as a tumor suppressor. It plays a negative role in the proliferation, invasion, and drug resistance of HCC cells and a vital role in the outcomes of patients with HCC [27]. To date, only a few studies have shown that FBXW7 interferes with the regulation of CSCs or tumor initiation cells, such as colorectal CSCs, gastric CSCs, and non-small-cell lung CSCs, and has a negative correlation with clinical recurrence and chemotherapy resistance [43–46]. Emerging evidence supported the existence of LCSCs and showed that HCC rapidly develops distant metastasis and resistance to drug therapies, at least partially due to the tumorigenicity and stemness characteristics acquired by the fractions of HCC cells [47]. However, the function and mechanism of FBXW7 in stem cell-like properties of HCC are unclear; hence, this study mainly focused on the stemness of HCC.

In a previous study, we applied a tissue microarray and IHC in 102 pairs of tumor and adjacent noncancerous tissues and revealed that FBXW7 has remarkably lower expression in tumor samples. Furthermore, some studies confirmed that FBXW7 downregulation in tumor tissues is associated with poor outcomes and progression of HCC [27]. The present study is aimed at testing whether FBXW7 is a stemness regulator of HCC cells. Consistent with the expected results, we confirmed that the expression of FBXW7 in spheres derived from MHCC-97H and Huh7 cells was lower than that in the monolayer adherent cells. Further functional experiments also confirmed that FBXW7 inhibits the self-renewal ability, stem-like phenotype, cell proliferation, and tumorigenicity of HCC cells, and combined with sorafenib, it enhances the antitumor properties. Therefore, we speculated that FBXW7 acts as an inhibitory factor and regulates the stemness of HCC. These results also lay a foundation to further explore the molecular mechanism of FBXW7 inhibiting the stem cell-like phenotype of HCC. To elucidate the underlying mechanism, mass spectrometry analysis and Co-IP experiments were used to forecast the substrates of FBXW7. ACTL6A, as a downstream target with a higher predictive score, was screened out for follow-up experiments. Accumulating evidence indicated that ACTL6A was dysregulated in various tumors, including HCC. The aberrant expression of ACTL6A was related to the development and had a prognosis value for HCC [48]. Consistent with this theory, patients with higher ACTL6A expression had poorer survival outcomes, based on the Kaplan–Meier plotter database. Moreover, functional experiments suggested that ACTL6A significantly increased the self-renewal capacity of the cells, tumorigenicity, and the expression of pluripotent transcription factors in MHCC-97H and Huh7 cells compared to the control cells. Based on the above experimental results, we speculated that the promoting effect of ACTL6A on the malignant phenotype and stemness of HCC cells might contribute to the poor outcomes of HCC patients.

To further assess the interaction between FBXW7 and ACTL6A in HCC, we investigated its relevance using proteomics. Protein ubiquitin modification is a regulatory mode of protein posttranslational modification that affects diverse biological processes [49]. Reportedly, FBXW7 regulates various biological processes by affecting the degradation of specific substrate proteins. Additional studies are required to test whether ACTL6A is the substrate and regulated by FBXW7-mediated proteasome degradation. First, we found that FBXW7 inhibited the expression of ACTL6A protein, while the level of *ACTL6A* mRNA is not altered in FBXW7-overexpressing cells. Furthermore, results showed that FBXW7 affects ACTL6A protein stability via the ubiquitination pathway. The results suggested that ACTL6A not only physically binds to FBXW7, but its function may be regulated by FBXW7-mediated ubiquitin and degradation. Next, we found that FBXW7 attenuated the effects of ACTL6A on HCC chemotherapy resistance. Therefore, we hypothesized that FBXW7 targets ACTL6A degradation in a ubiquitination-dependent manner. However, we are uncertain whether genotypic alterations in *FBXW7* genes play a central role in ACTL6A regulation. A study based on the standard coding sequence of various types of tumors showed that the total mutation rate of *FBXW7* in tumors was 6%, making it the fourth most common mutation gene after *TP53*, *KRAS*, and *APC* [50]. Reportedly, that FBXW7 regulates the biology of intestinal tumors by regulating the abundance of different substrates in a dose-dependent manner. FBXW7 haploinsufficiency fails to antagonize the Notch activity in human intestinal tumors. When biallelic inactivation occurs in FBXW7, it increases cellular proliferation in a c-Jun-dependent manner [42]. Hence, further in-depth investigation is warranted to elucidate the postoperative recurrence and insensitivity to multiple therapies in patients with HCC is accompanied by FBXW7 heterozygous or homozygous mutations.

In summary, the current study identified a novel FBXW7-ACTL6A axis and revealed its functions in stem cell-like properties in HCC. Importantly, we demonstrated that FBXW7 inhibited stemness by suppressing ACTL6A expression via the ubiquitination-dependent pathway. This phenomenon highlighted the importance of ACTL6A as a potential therapeutic target in human HCC with the absence of FBXW7. These findings elucidated the biological characteristics and regulatory mechanism of CSCs and provide new therapeutic strategies in cancer.

## Data Availability

The data used to support the findings of this article are available from the corresponding author upon request.

## Supplementary Materials

Supplementary Materials Supplementary Materials See Additional file 1 in Supplementary Materials for PCR primer sequences and immunoprecipitation-mass spectrometry analysis results.
