# Supplementary material for: FBXW7 Reduces the Cancer Stem Cell-Like Properties of Hepatocellular Carcinoma by Regulating the Ubiquitination and Degradation of ACTL6A
Source: Stem Cells Int. 2022 Sep 14;2022:3242482. doi: 10.1155/2022/3242482 (PMC9492413; doi:10.1155/2022/3242482)
Supplement: Supplementary Materials — See Additional file 1 in Supplementary Materials for PCR primer sequences and immunoprecipitation-mass spectrometry analysis results. [file 3242482.f1.docx]

Supplementary table.1 Sequences of qPCR primers used for mRNA analysis

| mRNA | Sequences | |
| --- | --- | --- |
| GAPDH | Forward | 5´-TGACTTCAACAGCGACACCCA-3´ |
|  | Reverse | 5´-CACCCTGTTGCTGTAGCCAAA-3´ |
| FBXW7 | Forward | 5´-GTCCCGAGAAGCGGTTTGGATA-3´ |
|  | Reverse | 5´-TGCTCAGGCACGTCAGAAAAG-3´ |
| Nanog | Forward | 5´-AACATGCAACCTGAAGACG-3´ |
|  | Reverse | 5´-CTATGAGGGATGGGAGGAG-3´ |
| SOX2 | Forward | 5´-CATCCACACTCACGCAAA-3´ |
|  | Reverse | 5´-CTCCCCAGGTTTTCTCTGT-3´ |
| OCT4 | Forward | 5´-GCAAGCCCTCATTTCACC-3´ |
|  | Reverse | 5´-CCATCACCTCCACCACCT-3´ |
| c-Myc | Forward | 5´-GCGGTCACACCCTTCTC-3´ |
|  | Reverse | 5´-CCGCTCCACATACAGTCC-3´ |

Supplementary table.2 The list of FBXW7 binding proteins identified by Immunoprecipitation-Mass Spectrometry

| ID | Accession | Gene Symbol | pI | #of Unique Peptides | Coverage (%) |
| --- | --- | --- | --- | --- | --- |
| 1 | sp\|O00159\|MYO1C_HUMAN | MYO1C | 9.9 | 9 | 13.8 |
| 2 | sp\|O00231\|PSD11_HUMAN | PSMD11 | 6.4 | 2 | 5.9 |
| 3 | sp\|O00571\|DDX3X_HUMAN | DDX3X | 7.2 | 7 | 11.6 |
| 4 | sp\|O96019\|ACL6A_HUMAN | ACTL6A | 5.3 | 2 | 10.3 |
| 5 | sp\|O75083\|WDR1_HUMAN | WDR1 | 6.6 | 3 | 8.3 |
| 6 | sp\|O95782\|AP2A1_HUMAN | AP2A1 | 7.0 | 18 | 25.8 |
| 7 | sp\|O96008\|TOM40_HUMAN | TOMM40 | 7.3 | 2 | 7.2 |
| 8 | sp\|P02545\|LMNA_HUMAN | LMNA | 7.0 | 44 | 54.5 |
| 9 | sp\|P04075\|ALDOA_HUMAN | ALDOA | 8.2 | 4 | 25.8 |
| 10 | sp\|Q6ZVX7\|FBX50_HUMAN | NCCRP1 | 6.6 | 1 | 10.9 |
| 11 | sp\|P08708\|RS17_HUMAN | RPS17 | 10.5 | 3 | 35.6 |
| 12 | sp\|Q9NXV2\|KCTD5_HUMAN | KCTD5 | 6.1 | 3 | 29.1 |
| 13 | sp\|Q03135\|CAV1_HUMAN | CAV1 | 5.9 | 3 | 28.7 |
| 14 | sp\|Q9UBP9\|GULP1_HUMAN | GULP1 | 8 | 3 | 14.1 |
| 15 | sp\|P32929\|CGL_HUMAN | CTH | 6.7 | 3 | 10.9 |
| 16 | sp\|Q86UA1\|PRP39_HUMAN | PRPF39 | 5.1 | 3 | 3.9 |
| 17 | sp\|P84095\|RHOG_HUMAN | RHOG | 8.2 | 2 | 20.9 |
| 18 | sp\|P30044\|PRDX5_HUMAN | PRDX5 | 9 | 2 | 20.6 |
| 19 | sp\|Q07021\|C1QBP_HUMAN | C1QBP | 4.5 | 2 | 14.5 |
| 20 | sp\|Q8N5Z5\|KCD17_HUMAN | KCTD17 | 4.5 | 2 | 14 |
| 21 | sp\|Q14681\|KCTD2_HUMAN | KCTD2 | 4.9 | 2 | 9.5 |
| 22 | sp\|Q9Y224\|RTRAF_HUMAN | RTRAF | 6.6 | 2 | 9.4 |
| 23 | sp\|Q13610\|PWP1_HUMAN | PWP1 | 4.4 | 2 | 9.2 |
| 24 | sp\|O15143\|ARC1B_HUMAN | ARPC1B | 8.4 | 2 | 8.9 |
| 25 | sp\|Q92804\|RBP56_HUMAN | TAF15 | 8.2 | 2 | 8.6 |
| 26 | sp\|P36873\|PP1G_HUMAN | PPP1CC | 6.5 | 2 | 7.4 |
| 27 | sp\|Q5T749\|KPRP_HUMAN | KPRP | 8.3 | 2 | 6.7 |
| 28 | sp\|P55209\|NP1L1_HUMAN | NAP1L1 | 4.1 | 2 | 5.9 |
| 29 | sp\|Q9Y265\|RUVB1_HUMAN | RUVBL1 | 6.4 | 2 | 5.9 |
| 30 | sp\|P04083\|ANXA1_HUMAN | ANXA1 | 7.0 | 5 | 18.2 |
